# Establishment of a novel diagnostic test algorithm for human T-cell leukemia virus type 1 infection with line immunoassay replacement of western blotting: a collaborative study for performance evaluation of diagnostic assays in Japan

**DOI:** 10.1186/s12977-020-00534-0

**Published:** 2020-08-24

**Authors:** Kazu Okuma, Madoka Kuramitsu, Toshihiro Niwa, Tomokuni Taniguchi, Yumiko Masaki, Gohzoh Ueda, Chieko Matsumoto, Rieko Sobata, Yasuko Sagara, Hitomi Nakamura, Masahiro Satake, Kiyonori Miura, Naoki Fuchi, Hideaki Masuzaki, Akihiko Okayama, Kazumi Umeki, Yoshihisa Yamano, Tomoo Sato, Masako Iwanaga, Kaoru Uchimaru, Makoto Nakashima, Atae Utsunomiya, Ryuji Kubota, Kenji Ishitsuka, Hiroo Hasegawa, Daisuke Sasaki, Ki-Ryang Koh, Mai Taki, Kisato Nosaka, Masao Ogata, Isao Naruse, Noriaki Kaneko, Sara Okajima, Kenta Tezuka, Emi Ikebe, Sahoko Matsuoka, Kazuo Itabashi, Shigeru Saito, Toshiki Watanabe, Isao Hamaguchi

**Affiliations:** 1 Department of Safety Research on Blood and Biological Products, National Institute of Infectious Diseases, Tokyo, Japan; 2 Research and Development Division, Fujirebio Inc., Tokyo, Japan; 3 Protein Technology, Engineering 1, Sysmex Corporation, Kobe, Japan; 4 Roche Diagnostics K.K., Tokyo, Japan; 5 Abbott Japan LLC, Tokyo, Japan; 6 Central Blood Institute, Blood Service Headquarters, Japanese Red Cross Society, Tokyo, Japan; 7 Department of Quality, Japanese Red Cross Kyushu Block Blood Center, Fukuoka, Japan; 8 Department of Obstetrics and Gynecology, Nagasaki University Graduate School of Biomedical Sciences, Nagasaki, Japan; 9 Department of Rheumatology, Infectious Diseases and Laboratory Medicine, University of Miyazaki, Miyazaki, Japan; 10 Department of Medical Life Science, Kyushu University of Health and Welfare, Miyazaki, Japan; 11 Division of Neurology, Department of Internal Medicine, St. Marianna University School of Medicine, Kawasaki, Japan; 12 Department of Rare Diseases Research, Institute of Medical Science, St. Marianna University School of Medicine, Kawasaki, Japan; 13 Department of Clinical Epidemiology, Nagasaki University Graduate School of Biomedical Sciences, Nagasaki, Japan; 14 Department of Computational Biology and Medical Sciences, Graduate School of Frontier Sciences, The University of Tokyo, Tokyo, Japan; 15 Department of Hematology and Oncology, Research Hospital, Institute of Medical Science, The University of Tokyo, Tokyo, Japan; 16 Department of Hematology, Imamura General Hospital, Kagoshima, Japan; 17 Division of Neuroimmunology, Joint Research Center for Human Retrovirus Infection, Kagoshima University, Kagoshima, Japan; 18 Department of Hematology and Rheumatology, Kagoshima University, Kagoshima, Japan; 19 Department of Laboratory Medicine, Nagasaki University Hospital, Nagasaki, Japan; 20 Department of Hematology, Osaka General Hospital of West Japan Railway Company, Osaka, Japan; 21 Rakuwakai Kyoto Medical Examination Center, Kyoto, Japan; 22 Department of Hematology, Kumamoto University of Medicine, Kumamoto, Japan; 23 Department of Hematology, Oita University Hospital, Oita, Japan; 24 Department of Infection and Immunology, SRL Inc., Tokyo, Japan; 25 Department of Pediatrics, Showa University School of Medicine, Tokyo, Japan; 26 Department of Obstetrics and Gynecology, University of Toyama, Toyama, Japan; 27 Department of Practical Management of Medical Information, St. Marianna University Graduate School of Medicine, Kawasaki, Japan

**Keywords:** HTLV-1 infection, HTLV-1 antibody, Diagnostic algorithm, Confirmatory test, WB, LIA, PCR

## Abstract

**Background:**

The reliable diagnosis of human T-cell leukemia virus type 1 (HTLV-1) infection is important, particularly as it can be vertically transmitted by breast feeding mothers to their infants. However, current diagnosis in Japan requires a confirmatory western blot (WB) test after screening/primary testing for HTLV-1 antibodies, but this test often gives indeterminate results. Thus, this collaborative study evaluated the reliability of diagnostic assays for HTLV-1 infection, including a WB-based one, along with line immunoassay (LIA) as an alternative to WB for confirmatory testing.

**Results:**

Using peripheral blood samples from blood donors and pregnant women previously serologically screened and subjected to WB analysis, we analyzed the performances of 10 HTLV-1 antibody assay kits commercially available in Japan. No marked differences in the performances of eight of the screening kits were apparent. However, LIA determined most of the WB-indeterminate samples to be conclusively positive or negative (an 88.0% detection rate). When we also compared the sensitivity to HTLV-1 envelope gp21 with that of other antigens by LIA, the sensitivity to gp21 was the strongest. When we also compared the sensitivity to envelope gp46 by LIA with that of WB, LIA showed stronger sensitivity to gp46 than WB did. These findings indicate that LIA is an alternative confirmatory test to WB analysis without gp21. Therefore, we established a novel diagnostic test algorithm for HTLV-1 infection in Japan, including both the performance of a confirmatory test where LIA replaced WB on primary test-reactive samples and an additional decision based on a standardized nucleic acid detection step (polymerase chain reaction, PCR) on the confirmatory test-indeterminate samples. The final assessment of the clinical usefulness of this algorithm involved performing WB analysis, LIA, and/or PCR in parallel for confirmatory testing of known reactive samples serologically screened at clinical laboratories. Consequently, LIA followed by PCR (LIA/PCR), but neither WB/PCR nor PCR/LIA, was found to be the most reliable diagnostic algorithm.

**Conclusions:**

Because the above results show that our novel algorithm is clinically useful, we propose that it is recommended for solving the aforementioned WB-associated reliability issues and for providing a more rapid and precise diagnosis of HTLV-1 infection.

## Background

Human T-cell leukemia virus type 1 (HTLV-1), a Deltaretrovirus genus member of the Retroviridae family, has a nonsegmented, positive-stranded RNA genome [1, 2]. HTLV-1 infection is endemic in south-west Japan, southern USA, the Caribbean, Jamaica, South America, central Australia, and equatorial Africa [3]. Although most HTLV-1-infected individuals, namely carriers, are asymptomatic, in some carriers HTLV-1 causes adult T-cell leukemia [4], HTLV-1-associated myelopathy/tropical spastic paraparesis [5], HTLV-1 uveitis [6], and other miscellaneous inflammatory manifestations [7] after long latent infection periods. HTLV-1 infects humans via three main routes: mother-to-infant transmission (vertical infection), which occurs mostly via breast-feeding, sexual transmission (horizontal infection), and blood transfusion [8–10]. A 2012 national survey in Japan reported a figure of around one million and eighty thousand asymptomatic Japanese carriers, which was 10% lower than that reported in 1988 [11], indicating that the total number of carriers has gradually decreased over time. However, it was reported in 2016 that over four thousand new infections have occurred in adolescent and adult blood donors in Japan [12], suggesting that further measures against horizontal infection, including the promotion of diagnostic tests for the infection, are urgently needed.

HTLV-1 infection is now routinely diagnosed by serological assays to detect HTLV-1 antibodies in Japan as follows. Peripheral blood from the subjects of interest is first screened by one of the following primary tests involving a diagnostic assay kit: particle agglutination (PA), chemiluminescent enzyme immunoassay (CLEIA), chemiluminescent immunoassay (CLIA), or electrochemiluminescence immunoassay (ECLIA), and non-reactive/negative results are diagnosed as no infection. However, the samples that test reactive by these methods are next examined by confirmatory western blot (WB) tests based on World Health Organization criteria [13, 14], because some may be false-positives. Following the results of both tests, the samples are finally judged to be definitely positive (determined infection), definitely negative (no infection), and indeterminate (undetermined infection). However, WBs often judge reactively screened samples to be indeterminate [15, 16], which is a major obstacle for accurate diagnosis of the infection [17, 18], and for whether breast feeding should be discouraged in mothers with indeterminate diagnoses to prevent the spread of infection in Japan [19, 20].

Recently, the line immunoassay (LIA) [21], which has been mainly used in Europe and Brazil as an alternative confirmatory test for HTLV-1 antibodies [22–25], has begun to be used in Japan [26]. Therefore, in this study, we sought to determine the current issues relating to a WB-based HTLV-1 diagnostic assay kit for Japanese samples, and to investigate the usefulness of the LIA as compared to WB for confirmation of sample reactivity. To achieve these goals we first analyzed and evaluated the performances of 10 commercially available diagnostic assay kits for HTLV-1 antibodies including WB and LIA, from four manufacturers using peripheral blood samples from blood donors and pregnant women in Japan. Based on the results obtained from these tests, we established a novel Japanese diagnostic test algorithm for HTLV-1 infection, which includes LIA in place of WB as an alternative confirmatory test, followed by nucleic acid detection using a previously standardized polymerase chain reaction (PCR) analysis [27–30] as an additional confirmatory test. We finally checked whether this algorithm was able to improve upon the frequent indeterminate results from WB analysis to make it a more clinically reliable diagnostic tool.

## Results

### Accuracy of the HTLV-1 diagnostic serological assay kits available in Japan

To assess the current issues seen with the WB-containing HTLV-1 diagnostic assay kits available in Japan, we evaluated the performances of 10 commercially available HTLV-1 antibody-specific diagnostic assay kits that include WB and LIA, from four manufacturers, using Japanese samples. First, to analyze the accuracy of the diagnostic serological assay kits, 50 conclusively HTLV-1-positive (primary detection-reactive and WB-positive) and 50 conclusively HTLV-1-negative (primary detection-negative) samples from blood donors were collected and tested with these assay kits.

**Fig. 1:**
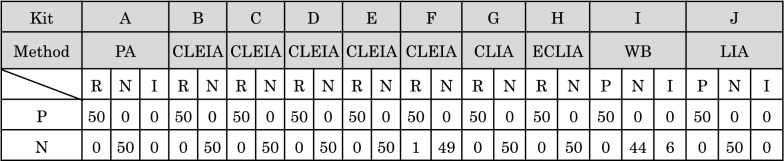
Accuracy testing of HTLV-1 diagnostic serological assay kits available in Japan on samples verified as positive or negative for HTLV-1. To evaluate the performances of the HTLV-1 diagnostic assay kits commercially available in Japan for HTLV-1 antibodies, 50 samples definitively positive for HTLV-1 antibodies and 50 samples definitively negative for HTLV-1 antibodies were tested with the 10 assay kits (kit **A:** PA, **B**–**F:** CLEIA, **G:** CLIA, **H:** ECLIA, **I:** WB, and **J:** LIA) listed in Table 1, according to the manufacturers' instructions. The number of HTLV-1-reactive, -positive, -negative or -indeterminate results determined by each kit is shown. Based on these results, the performance accuracy of each kit used was assessed. R: reactive, P: positive, N: negative, and I: indeterminate

As shown in Fig. 1, all the assay kits determined 50 of 50 conclusively positive samples to be positive, a 100% accuracy rate, whereas 8 assay kits except 2 kits (kits F and I) determined 50 of 50 conclusively negative samples to be negative, a 100% accuracy rate. However, kit F (with CLEIA) determined 49 of 50 conclusively negative samples to be negative, with 1 of 50 conclusively negative samples testing reactive, a 98.0% accuracy rate. Additionally, kit I (with WB) determined 44 of 50 conclusively negative samples to be negative, but 6 of 50 conclusively negative samples to be indeterminate, an 88.0% accuracy rate. These inconsistent results were most likely false positive or indeterminate results because all the other eight assay kits, including kit J (with LIA), exhibited the same negative results as expected.

These results indicate that the performances of almost all the diagnostic serological assay kits used for screening/primary testing were highly accurate, but the necessity of confirmatory testing to clear false positive results remains. These results also raise a serious issue with the performance of WB when used to confirm test results, as has been pointed out previously [15–18].

### Performances of HTLV-1 diagnostic serological assay kits on samples with indeterminate WB results

**Fig. 2:**
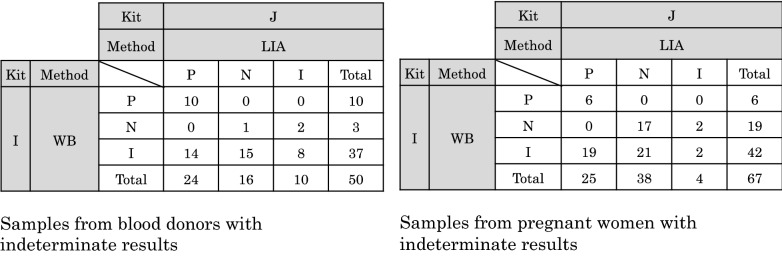
Performance evaluation of HTLV-1 diagnostic serological assay kits available in Japan on samples with indeterminate WB results. To further evaluate the performances of the HTLV-1 diagnostic serological assay kits commercially available in Japan, primary detection-reactive and WB-indeterminate samples from 50 blood donors and 67 pregnant women were tested with the same 10 assay kits used in the experiment for Fig. 1, according to the manufacturers' instructions. The results for each kit used are shown in Additional file 1: Figure S1. Herein, summary of the number of samples obtained and scored by kits I and J (WB and LIA, respectively) is exhibited. The performances of each of these kits were compared to determine their utility as confirmatory tests (left panel: blood donors, right panel: pregnant women). P: positive, N: negative, and I: indeterminate

To further evaluate the performances of the HTLV-1 diagnostic serological assay kits and, notably, to compare the usefulness of LIA in comparison with WB analysis, we tested samples from 50 blood donors and 67 pregnant women to confirm their primary detection-reactivity and WB-indeterminate status using these kits (Fig. 2 and Additional file 1: Figure S1).

As shown in Additional file 1: Figure S1, eight of the assay kits used for screening (kits A–H) produced similar results and modest sensitivity differences. These differences suggest that it is important to choose reagents with high sensitivity for HTLV-1 in the primary detection setting based on the most up-to-date information. Additionally, although all the tested samples were originally primary detection-reactive and WB-indeterminate, some screening kits produced some negative results, indicating that some of the negative samples might have been borderline for HTLV-1 when they were collected in terms of the detection sensitivity of the screening kits.

Nevertheless, Fig. 2 and Additional file 1: Figure S1 show that WB-based kit I produced many indeterminate results from 37/50 blood donors and 42/67 pregnant women (79/117, 67.5%), while 13/50 blood donors and 25/67 pregnant women were positive or negative (38/117, 32.5% decision rate). However, surprisingly, the LIA-based kit J judged only 10/50 blood donors and 4/67 pregnant women to be indeterminate (14/117, 12.0%), whereas 40/50 blood donors and 63/67 pregnant women were judged to be positive or negative (103/117, 88.0% decision rate). Among the 79 aforementioned indeterminate samples (37 blood donors and 42 pregnant women) re-judged by WB, 69 samples (29 blood donors and 40 pregnant women) tested LIA-positive or negative (87.3%: 78.4% and 95.2% decision rates, respectively). These results are important in showing that LIA significantly improves the judgment call on WB-indeterminate samples, leading to a more accurate diagnosis, making LIA more useful than WB for confirming serological reactivity.

Despite the considerable biological and immunological changes that occur within the body during pregnancy, there appeared to be no marked difference in detection judgments with any of the test methods used herein between blood donors and pregnant women.

### Serological sensitivity of LIA on provirus-positive samples with WB-indeterminate results

Because LIA showed a high decision rate for samples judged to be WB-indeterminate (as described above), we next assessed the serological sensitivity of LIA compared with that of WB on primary detection-reactive and WB-indeterminate samples with provirus positivity, as determined by PCR (“Methods” section), in more detail.

**Fig. 3:**
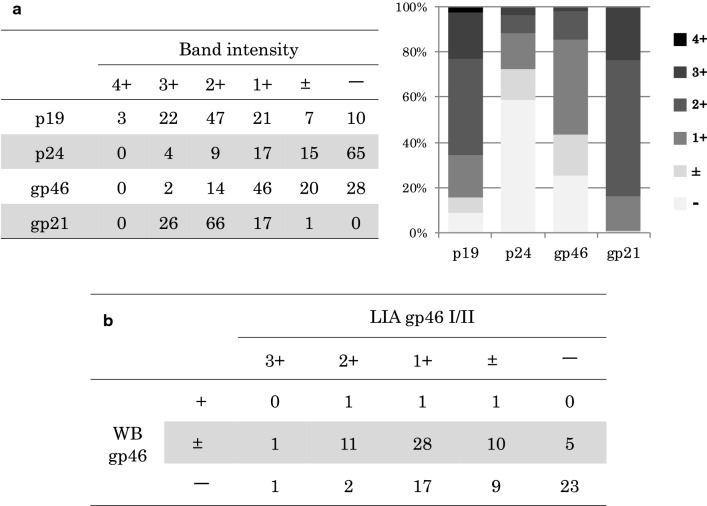
Serological sensitivity comparison between WB and LIA on WB-indeterminate samples with provirus positivity. To examine the performance of LIA as an alternative confirmatory test in more detail, 110 primary detection-reactive and WB-indeterminate samples with provirus positivity were subjected to LIA. **a** HTLV-1 antibody sensitivity to p19, p24, gp46, and gp21 of LIA was investigated. Samples that reacted or did not with each antigen were classified according to the band intensity from (−) to (4+) as indicated and depicted in the bar graph. **b** HTLV-1 antibody sensitivity to gp46 I/II (LIA test) and gp46 (WB test) was evaluated. Reactive/non-reactive samples were classified according to their band intensities from (−) to (3+) and from (−) to (+), respectively, and compared with each other

Among 110 primary detection-reactive and WB-indeterminate samples with provirus positivity, LIA judged 106 (96.4%) to be positive and 4 (3.6%) to be indeterminate (Additional file 2: Table S1). Importantly, 109 samples (99.1%) produced band intensities of ≧ (1+) with gp21 by LIA, indicating the high sensitivity of LIA against gp21 as compared with that against p19, p24, or gp46 (Fig. 3a). In addition, among the 107 samples showing (±) or (−) with gp46 by WB, 47 samples (43.9%) were also (±) or (−) with gp46 by LIA (Fig. 3b). These data show that LIA can clearly distinguish HTLV-1 infection in carriers with indeterminate WB results by its stronger serological sensitivity, which led to a marked reduction in indeterminate judgment calls. This also suggests that because the difference in sensitivity to gp46 between WB and LIA was intermediate, gp46 may not be a robust target protein for diagnostic serological testing for HTLV-1 antibodies.

These results provide supporting evidence for the usefulness of LIA as an alternative confirmatory test to WB-based detection, and for LIA to replace WB as the next-generation diagnostic algorithm for HTLV-1 infection.

### Establishment of a novel diagnostic test algorithm for HTLV-1 infection in Japan

**Fig. 4:**
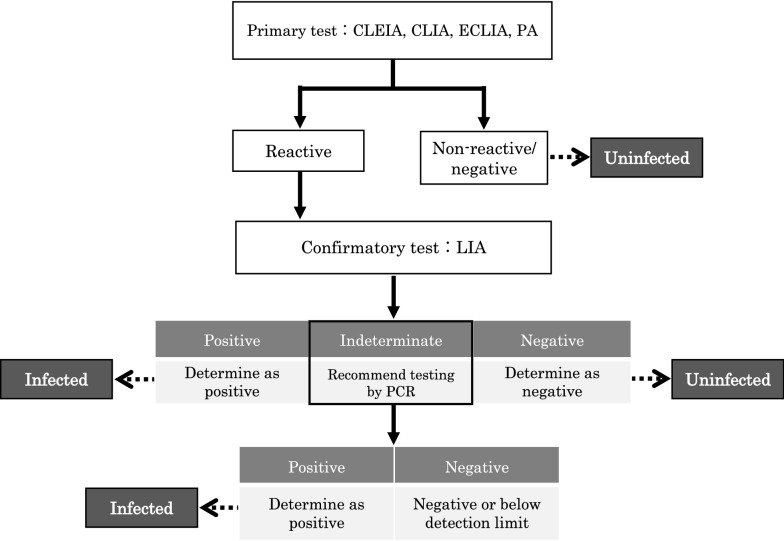
Flowchart of our newly established test algorithm for diagnosing HTLV-1 infection in Japan. Based on the results obtained in the performance evaluation of the HTLV-1 diagnostic serological assay kits commercially available in Japan, we established a novel HTLV-1 diagnostic test algorithm for use in Japan. This test algorithm includes a primary test with CLEIA, CLIA, ECLIA, or PA, a confirmatory test with LIA but not WB, and an additional confirmatory test with PCR when samples test indeterminate by LIA. How to judge the output of the test results and how to determine the infection status of a sample using the algorithm are shown on the flowchart

Based on assessing the results obtained from the aforementioned serological assays, we established a novel diagnostic test algorithm for HTLV-1 infection in Japan (Additional file 4), whereby diagnostic testing for HTLV-1 and its determination should be performed according to the flowchart in Fig. 4, and as described below.

First, primary detection testing/screening on the serum or plasma obtained from peripheral blood samples is conducted to determine the HTLV-1 infection status. Commercially available HTLV-1 antibody-specific serological assays (PA, CLEIA, CLIA, and ECLIA) are recommended as the primary detection methods. Determining the primary detection test results and subsequent measures should proceed as follows: (i) When “non-reactive/negative”, an uninfected status will be definitely determined. (ii) When “reactive”, confirmatory testing will always be performed for a definitive diagnosis. Exceptionally, when the PA is “indeterminate”, re-testing by the PA or another primary detection method will be recommended to obtain the “non-reactive/negative” or “reactive” result.

Next, for confirmation, we recommend testing for HTLV-1 antibodies using LIA (but not WB) and conducting HTLV-1 PCR [28, 29] when the result is indeterminant with LIA. (i) When “positive”, a diagnosis will be established of HTLV-1 infection (infectious disease). (ii) When “negative”, a diagnosis will be established as negative for HTLV-1 infection (no infectious disease). (iii) With “indeterminant” samples, if the PCR is “positive”, a diagnosis of HTLV-1 infection is appropriate. If PCR testing is “negative”, a negative or below the limit of detection (< 4 copies/10^5^ cells) [29] result is appropriate.

### Clinical suitability of the newly established diagnostic test algorithm for HTLV-1 infection

**Fig. 5:**
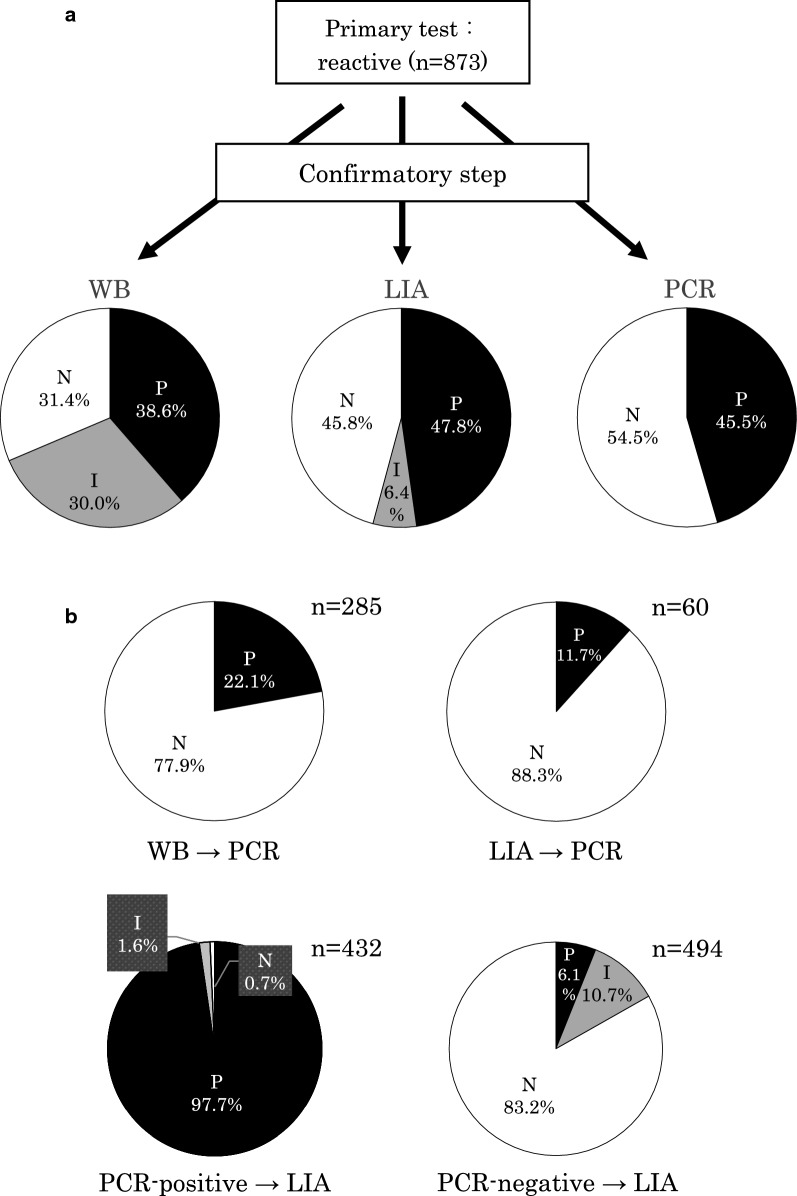
Clinical evaluation of our newly established diagnostic test algorithm for HTLV-1 infection. The suitability of the established HTLV-1 diagnostic test algorithm was evaluated clinically. **a** Eight hundred and seventy-three samples determined reactive by a primary test were in parallel confirmed for positivity by WB, LIA, and PCR at each domestic clinical laboratory. The rates of each judgment were calculated and are indicated as percentages in the pie graphs. **b** To determine the best test algorithm, 285 WB-indeterminate samples, 60 LIA-indeterminate samples, 432 PCR-positive samples, and 494 PCR-negative samples, which were all primary test-reactive, were additionally (secondly) confirmed by PCR or LIA on the former two and the latter two samples, respectively, at each domestic clinical laboratory. Herein the rates of each re-judgment by PCR or LIA were calculated, as indicated by percentages (upper and lower pie graphs, respectively). P: positive, N: negative, and I: indeterminate

We finally evaluated the clinical suitability of the novel HTLV-1 diagnostic test algorithm established above. Eight hundred and seventy-three samples determined to be reactive by primary testing were tested in parallel by WB, LIA, and PCR, and the reactivity status of these samples was confirmed at each domestic clinical laboratory (Fig. 5a). As a result, the decision rates for the confirmatory testing by WB, LIA, and PCR were 70.0% (611/873: 337/873, 38.6% positive; 274/873, 31.4% negative), 93.6% (817/873: 417/873, 47.8% positive; 400/873, 45.8% negative), and 45.5% (397/873, 45.5% positive; 476/873, 54.5% negative), respectively, again indicating the much better performance of LIA over WB.

To assess the usefulness of PCR for the additional (second) confirmation as indicated in the algorithm, 285 WB-indeterminate samples and 60 LIA-indeterminate samples were tested and then subjected to PCR confirmation (Fig. 5b upper graphs). Consequently, PCR testing on the WB-indeterminate samples (PCR following WB) and PCR on the LIA-indeterminate samples (PCR following LIA) produced a 22.1% (63/285) positive decision rate and 11.7% (7/60) positive decision rate, respectively. These data show that the WB-indeterminate samples contained more positive samples than the LIA-indeterminate samples, indicating the aforementioned WB-associated reliability issue again and the usefulness of PCR as an additional confirmatory test.

Because PCR produced a similar positivity rate to that of LIA (45.5% vs 47.8%, respectively) as described above, we next examined the performance of PCR in more detail. Four hundred and thirty-two PCR-positive samples and 494 PCR-negative samples were tested and confirmed by LIA (Fig. 5b lower graphs). LIA on the PCR-positive and the PCR-negative samples (LIA following PCR) had a 98.4% decision rate (425/432: 422/432, 97.7% positive; 3/432, 0.7% negative) and an 89.3% decision rate (441/494: 30/494, 6.1% positive; 411/494, 83.2% negative), respectively. These data show that although PCR positivity accords well with LIA positivity, as anticipated, the PCR-negative samples contained many LIA-positive ones (30/494, 6.1%, see above). This suggests that when used as a first confirmation, PCR may fail to capture many positive cases, leading to an unnecessary delay in diagnosis determination.

The results obtained herein reveal that confirmatory tests by LIA followed by PCR as a diagnostic test algorithm, is an efficient algorithm when compared with the other combinations assessed. These findings also indicate that the novel HTLV-1 diagnostic test algorithm established in this study may be suitable for clinical use and, if implemented, it should help to provide a quicker and more accurate diagnosis of HTLV-1 infection.

## Discussion

In Japan, as part of a government measure aimed at preventing mother-to-child transmission of HTLV-1, HTLV-1 antibody testing has been publicly funded for prenatal screening since 2010. In 2011, HTLV-1 antibody screening was changed to Recommendation Level A (highly recommended) for pregnant women in the Guideline for Gynecological and Obstetric Practice in Japan and, consequently, more pregnant women are now being tested for HTLV-1 antibodies.

HTLV-1 infection (an infectious disease) is diagnosed using a primary HTLV-1 antibody detection test, followed by detection based on WB analysis as confirmation for individuals who were reactive on the primary detection test. In recent years, both the sensitivity and specificity of the antibody detection method have improved. However, 10–20% of confirmatory assays are diagnosed as “indeterminate” in Japan, which is a major drawback to accurate diagnosis. One explanation for this may be related to low or no expression of viral antigen(s) resulting from by mutation(s) in such antigens in the HTLV-1 genome, leading to low levels of HTLV-1 antibodies and/or non-specific reactions [15, 16, 29, 31, 32]. Indeterminate judgment based on WB confirmation is a particular issue for pregnant women who would like to breast feed their infants; therefore, finding the best method to accurately determine the infection status of pregnant women is important.

The HTLV-1 PCR method that specifically detects HTLV-1 proviral DNA from peripheral blood cell genomes is known to be effective in determining HTLV-1 infection in such indeterminant cases, although a drawback to it has been the absence of a standard protocol for measuring HTLV-1 proviral DNA loads (PVLs) [33–35]. To address this problem, we established standardization protocols around 2014 [27–30] and, since 2016, PCR has been covered by health insurance in pregnant women considered indeterminate for HTLV-1 infection using WB testing in Japan.

In further efforts to solve the long-standing problem with the WB approach described above, we evaluated the LIA method, an immunoblot approach based on recombinant antigens and synthetic peptides from HTLV-1/2 Gag and Env proteins, as an alternative confirmatory test [21]. LIA is commonly used in Europe and Brazil, and has been reported to be useful for confirmatory testing of HTLV-1 infection [22–25]. Quite recently this assay started to be available in Japan also [26], but as HTLV-1 in Japan belongs to a Japanese subgroup within Cosmopolitan subtype A [36], the assessment using Japanese samples as an alternative confirmatory test of LIA was insufficient. Therefore, we in this collaborative study group performed a relatively large-scale assessment of diagnostic serological assays including LIA using Japanese samples. As a result, we determined that LIA is useful for confirmatory testing in Japan. LIA was approved for insurance coverage in 2017, and since 2018 pregnant women are also covered by health insurance in Japan for PCR testing when they have indeterminate LIA results.

The HTLV-1 *env* gene encodes Env, gp46 and gp21 envelope glycoproteins. These proteins, which are cleaved from the envelope precursor glycoprotein gp62, are responsible for the specific binding of HTLV-1 to cellular receptor(s) and catalyze virus–cell membrane fusion at the cell surface, resulting in viral entry into host cells [37]. The Env proteins are also immunodominant among HTLV-1 viral antigens [38]. In the present study, the antibody sensitivity against gp21 was even stronger than against other HTLV-1 antigens including gp46 by LIA, indicating that gp21 is currently the best antigen for detecting HTLV-1 antibodies.

The serological assay kits tested in this study (a part of CLEIAs, CLIA, ECLIA, and LIA) have now become available for detecting both HTLV-1 and HTLV-2 infections. However, HTLV‐2 pathogenesis is thought to be much rarer than that seen with HTLV‐1, although it is reported that HTLV‐2 is associated with a neurological disorder [39]. Thus, because HTLV‐1 and HTLV‐2 are quite different pathologically, an additional method to differentiate these infections is needed when LIA-positive samples are found to be HTLV-positive in the confirmatory testing. Therefore, we recently developed quantitative PCR to detect nucleic acids from HTLV‐1 and/or HTLV‐2 proviruses with high sensitivity [40], making such a discrimination possible.

Based on our recent and present findings, we established a new HTLV-1 detection algorithm/protocol including both LIA (instead of WB) and PCR, and showed the clinical suitability of this algorithm, namely LIA followed by PCR, in Japan, in the current study. However, because rare LIA negativity (0.7%) in PCR-positive cases was seen in Fig. 5b (Additional file 3: Table S2), there appeared to be a limitation on this algorithm, which in the future may possibly require the improvement including more sensitive kit(s) and/or repeated testing(s) as mentioned in the foreign guidance [41].

Thus, at the present time, we propose and recommend this diagnostic test algorithm as the “Diagnostic Guidelines for HTLV-1 infection in Japan” (Additional file 4). In doing this we aim to create an accurate HTLV-1 diagnostic guideline for wide application, based on the most recently tested methods, to be used at the earliest opportunity in Japan.

## Conclusions

In this collaborative study, we evaluated LIA as an alternative confirmatory test candidate to replace the current confirmatory WB test for HTLV-1 antibodies that frequently produces indeterminate results, using blood samples obtained in Japan. Our results show that LIA significantly reduces the occurrence of indeterminate results when compared with WB analysis of Japanese samples. Our newly established, novel diagnostic test algorithm for HTLV-1 infection in Japan proposes that LIA replaces WB as the first confirmatory test and that PCR should be used as the second confirmatory test. When we performed WB, LIA, and/or PCR in parallel as confirmatory tests at our clinical laboratories, the test algorithm that we established, namely, LIA followed by PCR was shown to be superior to the other methods tested for diagnosis. Because the usefulness of our novel algorithm has been clinically confirmed in Japan, we now propose and recommend this algorithm for diagnosing HTLV-1 infection more efficiently in Japan and hopefully in other countries where HTLV-1 is endemic.

## Methods

### HTLV-1 diagnostic serological assay kits

The performances of 10 diagnostic assay kits commercially available in Japan for detecting HTLV-1 antibodies, from four manufacturers, were assessed in this study (Table 1). The kits tested are as follows: (1) PA in Serodia^®^ HTLV-I (Fujirebio Inc., Tokyo, Japan); (2) CLEIA in Lumipulse^®^ HTLV-I, Lumipulse^®^ Presto HTLV-I, Lumipulse^®^ HTLV-I/II, Lumipulse^®^ Presto HTLV-I/II (Fujirebio Inc.), and HISCL HTLV-I Ab (Sysmex Co., Kobe, Japan); (3) CLIA in ARCHITECT^®^ HTLV (Abbott Japan LLC, Tokyo, Japan) [42]; (4) ECLIA in Elecsys^®^ HTLV-I/II (Roche Diagnostics K.K., Tokyo, Japan) [43]; (5) WB in Problot HTLV-I (Fujirebio Inc.) [13]; (6) LIA in INNO-LIA HTLVI/II Score (Fujirebio Inc.) [21]. Serological assays using these kits were performed according to the manufacturers’ instructions. The judgement criteria used for each kit are as follows: (1)  < 16× negative, ≧ 16× reactive, if 16X is (±) indeterminate; (2), (4) cut off index (C.O.I.)  < 1.0 negative, ≧ 1.0 reactive; (3) signal/cut-off (S/CO)  < 1.0 negative, ≧ 1.0 reactive. The criteria for kits (5) and (6) are described below.

**Table 1:** List of HTLV-1 diagnostic serological assay kits commercially available in Japan that were used in this study

Kit name	Method	Manufacturer
ARCHITECT^®^ HTLV	CLIA	Abbott Japan LLC
Elecsys^®^ HTLV-I/II	ECLIA	Roche Diagnostics K.K.
HISCL HTLV-I Ab	CLEIA	Sysmex Corporation
Serodia^®^ HTLV-I	PA	Fujirebio Inc.
Lumipulse^®^ HTLV-I	CLEIA	Fujirebio Inc.
Lumipulse^®^ Presto HTLV-I	CLEIA	Fujirebio Inc.
Lumipulse^®^ HTLV-I/II	CLEIA	Fujirebio Inc.
Lumipulse^®^ Presto HTLV-I/II	CLEIA	Fujirebio Inc.
Problot HTLV-I	WB	Fujirebio Inc.
INNO-LIA HTLV-I/II Score	LIA	Fujirebio Inc.

*CLIA* chemiluminescent immunoassay, *ECLIA* electrochemiluminescence immunoassay, *CLEIA* chemiluminescent enzyme immunoassay, *PA* particle agglutination, *WB* western blot, *LIA* line immunoassay

### Blood samples used for evaluating the performances of the diagnostic assay kits

To compare and evaluate the performance of HTLV-1 antibody assay kits, notably WB and LIA, WB-judged/confirmed samples including WB-indeterminate samples needed to be prepared in this experiment. Thus, when we tested and collected blood samples, we employed a current diagnostic routine in Japan: primary testing routinely used at each laboratory, followed by WB confirmation.

Peripheral blood was obtained from Japanese Red Cross blood donors in Kyushu and Tokyo, Japan, and from pregnant women at two hospitals in Nagasaki and Tokyo, Japan. Plasma derived from these samples was mostly screened by CLEIA with Lumipulse^®^ Presto HTLV-I (Fujirebio Inc.), and reactive samples were confirmed by WB analysis with Problot HTLV-I (Fujirebio Inc.), according to the manufacturer's instructions. Briefly, in the Problot HTLV-I, p19, p24, p53 bands for Gag and gp46 band for Env were used to interpret the results. The bands are defined as 3 grades; namely (−), (±), or (+), and when all the bands are (−), the result was judged to be negative. When Env gp46 and any of Gag p19, p24, or p53 are (+), the result was judged to be positive. Band patterns that match neither negative nor positive results were judged to be indeterminate.

Consequently, all the samples tested could be divided into the following three groups: 50 conclusively positive samples from blood donors, 50 conclusively negative samples from blood donors, and 117 indeterminate samples (50 from blood donors, 67 from pregnant women). These samples were used to evaluate the performances of the HTLV-1 diagnostic assay kits commercially available in Japan.

### Use of WB-indeterminate and provirus-positive blood samples for performance comparison between WB and LIA

Peripheral blood from WB-indeterminate blood donors was obtained from the Japanese Red Cross (Fukuoka, Japan) via CLEIA screening with Lumipulse^®^ Presto HTLV-I or Lumipulse^®^ Presto HTLV-I/II (Fujirebio Inc.) followed by WB confirmation using Problot HTLV-I (Fujirebio Inc.), as described above. Genomic DNA was purified from blood clots using the QIAsymphony DSP DNA midi kit (QIAGEN, Hilden, Germany). The HTLV-1 PVLs (copies/100 cells, %) in the genomic DNA samples were estimated by quantitative PCR to determine provirus positivity as previously reported [28, 29, 44]. Confirmatory LIA tests were performed on 110 CLEIA-reactive, WB-indeterminate, and provirus-positive blood samples with INNO-LIA HTLV-I/II Score (Fujirebio Inc.) according to the manufacturer's instructions. Briefly, four antigen lines (p19 and p24 I/II Gag, and gp46 and gp21 I/II Env) were assessed for their ability to detect HTLV-1/2 antibodies, and three antigen lines (p19 I Gag and gp46 I/II Env) were assessed for their ability to discriminate between HTLV-1 and HTLV-2 antibodies, as compared with control lines. The antibody sensitivity to each antigen was also evaluated based on the intensity of bands by comparison with that of the control antigen, according to the criteria of the manufacturer's instructions, and expressed as (−) to (4+).

Based on the performance (serological sensitivity) data, WB and LIA were compared. Data including CLEIA-specific antibody titers, the profiles of both WB and LIA blot patterns, and the PVLs in all the blood samples used in this experiment are listed in Additional file 2: Table S1.

### Clinical evaluation of the novel diagnostic algorithm developed herein by laboratory testing

To clinically evaluate the diagnostic test algorithm for HTLV-1 infection, as established in this study, and as described in the Results section, 873 primary detection-reactive blood samples were collected and confirmed by WB, LIA, and PCR in parallel at two domestic clinical laboratories (first confirmation). Another 285 primary detection-reactive WB-indeterminate and 60 primary detection-reactive LIA-indeterminate blood samples were collected and their test results were additionally confirmed by PCR, while another 432 primary detection-reactive PCR-positive and 494 primary detection-reactive PCR-negative blood samples were collected and additionally confirmed by LIA at four domestic clinical laboratories (second confirmation); that is, WB or LIA followed by PCR and PCR followed by LIA, respectively. These assays were performed as described above. From the data obtained the clinical values of the confirmatory testing algorithms were compared with each another and assessed.

## Supplementary information

**Additional file 1: Figure S1.** Performance evaluation of HTLV-1 diagnostic serological assay kits available in Japan on WB-indeterminate samples.

**Additional file 2: Table S1.** For performance (serological sensitivity) comparison between WB and LIA, 110 CLEIA-reactive, WB-indeterminate, and provirus-positive blood samples were collected and tested by LIA. CLEIA-specific antibody titers, the profiles of both WB and LIA blot patterns, the judgments by each test method, and the PVLs by PCR in all the blood samples used in this experiment are listed. NT: not tested, R: reactive, and I: indeterminate.

**Additional file 3: Table S2.** CLEIA-specific antibody titers, the profiles of LIA blot patterns, the judgments by each test method, and the PVLs by PCR in three primary test-reactive, LIA-negative, and PCR-positive blood samples found in Fig. 5b are listed. NT: not tested, R: reactive, and N: negative.

**Additional file 4.** Diagnostic Guidelines for Human T-Cell Leukemia Virus Type 1 Infection in Japan, Version 2 (November 2019).

## Data Availability

The data used in this study are available from the corresponding author based on reasonable request.

## Abbreviations

HTLV-1: human T-cell leukemia virus type 1
PA: particle agglutination
CLEIA: chemiluminescent enzyme immunoassay
CLIA: chemiluminescent immunoassay
ECLIA: electrochemiluminescence immunoassay
WB: western blot
LIA: line immunoassay
PCR: polymerase chain reaction
