# Supplementary material for: Establishment of a novel diagnostic test algorithm for human T-cell leukemia virus type 1 infection with line immunoassay replacement of western blotting: a collaborative study for performance evaluation of diagnostic assays in Japan
Source: Retrovirology. 2020 Aug 24;17:26. doi: 10.1186/s12977-020-00534-0 (PMC7444053; doi:10.1186/s12977-020-00534-0)
Supplement: Supplementary file 1 — Additional file 1: Figure S1. Performance evaluation of HTLV-1 diagnostic serological assay kits available in Japan on WB-indeterminate samples. [file 12977_2020_534_MOESM1_ESM.docx]

Figure S1

a

Figure S1

b

**Figure S1 Performance evaluation of HTLV-1 diagnostic serological assay kits available in Japan on WB-indeterminate samples.** Primary detection-reactive and WB-indeterminate samples from 50 blood donors (a) and 67 pregnant women (b) were tested with the same 10 assay kits (kit A: PA, B–F: CLEIA, G: CLIA, H: ECLIA, I: WB, and J: LIA) used in the experiment for Fig. 1, according to the manufacturers' instructions. The results for each kit used are shown. The judgment criteria for each kit used are as follows: kit A < 16X negative, ≧ 16X reactive, if 16X is (±) indeterminate; kits B–F, H cut off index (C.O.I.) < 1.0 negative, ≧ 1.0 reactive; kit G signal/cut-off (S/CO) < 1.0 negative, ≧ 1.0 reactive. The criteria for kits I and J are described in the Methods section. R: reactive, P: positive, N: negative, and I: indeterminate
