# Supplementary material for: Establishment of a novel diagnostic test algorithm for human T-cell leukemia virus type 1 infection with line immunoassay replacement of western blotting: a collaborative study for performance evaluation of diagnostic assays in Japan
Source: Retrovirology. 2020 Aug 24;17:26. doi: 10.1186/s12977-020-00534-0 (PMC7444053; doi:10.1186/s12977-020-00534-0)
Supplement: Supplementary file 4 — Additional file 4. Diagnostic Guidelines for Human T-Cell Leukemia Virus Type 1 Infection in Japan, Version 2 (November 2019). [file 12977_2020_534_MOESM4_ESM.docx]

**Diagnostic Guidelines**

**for Human T-Cell Leukemia Virus Type 1 Infection**

**in Japan**

**Version 2 (November 2019)**

**The 2019 Japan Agency for Medical Research and Development (AMED) subsidized research and development grant**

**Research project to promote the development of innovative drugs**

**for emerging and re-emerging infectious diseases**

**“Epidemiological study of HTLV-1 and research contributing**

**to comprehensive measures”**

The 2019 Japan Agency for Medical Research and Development (AMED)

subsidized research and development grant

Research project to promote the development of innovative drugs

for emerging and re-emerging infectious diseases

“Epidemiological study of HTLV-1 and research contributing

to comprehensive measures” group

Research representative: Isao Hamaguchi (National Institute of Infectious Diseases)

Research team/collaborators:

Toshiki Watanabe St. Marianna University School of Medicine, The University of Tokyo

Kaoru Uchimaru The University of Tokyo

Yoshihisa Yamano St. Marianna University School of Medicine

Masahiro Satake Japanese Red Cross Society

Yasuko Sagara Japanese Red Cross Society

Shigeru Saito University of Toyama

Atae Utsunomiya Imamura General Hospital

Ryuji Kubota Kagoshima University

Kenji Ishitsuka Kagoshima University

Akihiko Okayama University of Miyazaki

Kazumi Umeki University of Miyazaki

Masako Iwanaga Nagasaki University

Hiroo Hasegawa Nagasaki University

Hideaki Masuzaki Nagasaki University

Kiyonori Miura Nagasaki University

Masao Ogata Oita University

Kisato Nosaka Kumamoto University

Ki-Ryang Koh Osaka General Hospital of West Japan Railway

Company

Mai Taki Rakuwakai Kyoto Kenshin Center

Kazu Okuma National Institute of Infectious Diseases

Sahoko Matsuoka National Institute of Infectious Diseases

Madoka Kuramitsu National Institute of Infectious Diseases

1. **Introduction**

As a measure to prevent mother-to-child transmission of human T-cell leukemia virus type 1 (HTLV-1) in Japan, the HTLV-1 antibody test was added as one of the test items to the publicly subsidized health examination for pregnant women in November 2010, and in April 2011, the HTLV-1 antibody test for pregnant women was raised to Recommendation Level A (strongly recommended) in the obstetrics and gynecology practice guidelines in Japan. Presently, all applicants of pregnant women undergo HTLV-1 antibody test.

To date, to diagnose HTLV-1 infection HTLV-1 antibodies have been tested by a primary/screening test and western blot (WB) as a confirmatory test for individuals found reactive in the primary test. Recently, although the sensitivity and specificity of antibody tests have improved, the issue is that 10%–20% of WB tests return with a “indeterminate” result, and particularly, the issue of “indeterminate” results in pregnant women is related to the selection of breast-feeding methods; thus, an accurate confirmation (determination of the presence or absence) of infection is needed.

For “indeterminate” results in confirmatory tests, nucleic acid detection (polymerase chain reaction [PCR]) methods that specifically detect HTLV-1 viral DNA (proviral DNA) in the genomes of peripheral blood cells have been known to be helpful in establishing HTLV-1 infection; however, issues remained in which no standard protocols were established^1^. To address this issue, a standardization effort was completed by the Health, Labour and Welfare Sciences Research Group in 2014^2^, and the HTLV-1 nucleic acid detection (PCR) method was covered by insurance for pregnant women with “indeterminate” results of WB tests in April 2016. The research group also suggested that the line blot (line immunoassay [LIA]) method^footnote 1^ was useful as a confirmatory test, and testing by LIA has also been covered from November 1, 2017. Additionally, the HTLV-1 nucleic acid detection (PCR) method for pregnant women with “indeterminate” results via LIA has been covered from April 2018. Subsequently, sales and marketing for the WB kit in Japan were discontinued in March 2019.

Footnote 1. Method for detecting specific antibodies reacting with antigens through enzymatic reactions using a solid-phase membrane on which HTLV-1 and HTLV-2 recombinant antigens or synthetic peptide antigens are immobilized

Thus, the same research group decided to publish new recommended laboratory testing procedures as “diagnostic guidelines for HTLV-1 infection” based on current medical knowledge to ensure that guidance on the accurate diagnosis of HTLV-1 infection using latest laboratory testing methods is widely disseminated at an early stage, including guidance on using the LIA method as the only confirmatory test to replace the WB test and adding the HTLV-1 nucleic acid detection (PCR) method.

These diagnostic guidelines are based on information as of November 2019.

1. **Approach toward setting guidelines**

With advances in HTLV-1 antibody testing, in addition to antibody testing by particle agglutination (PA) and chemiluminescent enzyme immunoassay (CLEIA) methods that have been previously recommended as methods for primary testing in the Manual for Prevention of HTLV-1 Mother-to-Child Transmission and Guidance on Health (March 2011)^3^, chemiluminescent immunoassay (CLIA) and electrochemiluminescence immunoassay (ECLIA) methods were found to have comparable test accuracy to conventional tests^4-6^; thus, the addition of CLIA and ECLIA methods is recommended.

Moreover, when the Health, Labour and Welfare Sciences Research Group additionally performed HTLV-1 nucleic acid detection (PCR) methods for individuals with “indeterminate” results from WB confirmatory tests, which have been regarded as an issue to date, HTLV-1 provirus was detected, and in infected individuals with low antibody titers, a definitive diagnosis using the antibody detection system was difficult; it became clear that use of the HTLV-1 nucleic acid detection (PCR) method was effective^2^. Additionally, the same research group clarified that the rate of “indeterminate” results was greatly reduced using LIA as a confirmatory test. Furthermore, there were instances of reactive results in primary tests but negative results in confirmatory tests, indicating the importance of the confirmatory test.

Based on the aforementioned findings, for this diagnosis, it is recommended that a confirmatory LIA is performed whenever a primary test is reactive and an HTLV-1 nucleic acid detection (PCR) method is performed if “indeterminate” results are obtained from confirmatory tests.

1. **Practical diagnostic methods**
2. **Primary testing**
3. This test is performed via medical consultation to determine the presence of HTLV-1 infection.
4. As a diagnostic test, it is crucial to select products with sufficiently high sensitivity based on the latest information on HTLV-1 primary testing methods.
5. In addition to the previously recommended PA and CLEIA methods, CLIA and ECLIA methods are recommended for primary testing.
6. The results of the primary testing and subsequent steps will be as follows:
   1. If a negative result is obtained

At this point, an “uninfected (not infected)” diagnosis is confirmed.

- 1. If a reactive result is obtained

Confirmatory testing should always be performed to verify the results.

1. **Confirmatory testing**

In confirmatory testing, it is recommended that HTLV-1 antibody testing with LIA is performed, and if a “indeterminate” result is obtained from LIA, an HTLV-1 nucleic acid detection (PCR) method is performed. Testing and assessments should be performed according to the flowchart in the appendix.

1. If results for HTLV-1 antibodies via LIA are positive

Positive results are considered confirmed, and “HTLV-1 infection” is diagnosed.

1. If results for HTLV-1 antibodies via LIA are negative

Negative results are considered confirmed as “uninfected (not infected)”.

1. If results for HTLV-1 antibodies via LIA are “indeterminate”
2. If HTLV-1 nucleic acid detection (PCR) method results are positive, “HTLV-1 infection” is diagnosed.
3. If HTLV-1 nucleic acid detection (PCR) method results are negative, an “uninfected or below detection limit (<4 copies/10^5^ cells)^7^” result is considered confirmed.
4. **Points to consider**
5. **Points to consider regarding testing**
6. Many subjects do not know that they will not be diagnosed with HTLV-1 infection even if the primary test is reactive unless a positive confirmatory test result is obtained. Therefore, healthcare professionals must take the aforementioned concern into account when explaining results to individuals with a reactive primary test. Thus, if the results of the primary test are reactive, assurance must be obtained that the subject understands that “the results of the primary test are reactive, and infection will not be confirmed until the results of the confirmatory test are obtained.” Careful attention should be paid to healthcare professionals who provide explanations to avoid misleading or concerning explanations that “a reactive primary test indicates HTLV-1 infection.”
7. These guidelines are intended for use at the time of HTLV-1 infection diagnosis. Thus, the HTLV-1 nucleic acid detection (PCR) method set in the guidance is a qualitative test and is not intended to measure HTLV-1 proviral load (quantitative testing).
8. For HTLV-1 nucleic acid detection (PCR) methods, it is recommended that as much as 1 μg of genomic DNA is used to achieve adequate sensitivity as a confirmatory test^7^.
9. **Points to consider regarding maternal care**

Maternal practices after confirming HTLV-1 infection will be in accordance with the Manual for Prevention of HTLV-1 Mother-to-Child Transmission (March 2017)^8^.

However, if an HTLV-1 nucleic acid detection (PCR) method is performed during maternity care and the result is below detection limit (<4 copies/10^5^ cells), mother-to-child transmission is considered unlikely to occur^footnote 2^. Nevertheless, the safety of long-term breast-feeding of these clinical cases remains inconclusive.

Footnote 2. In previous studies, the likelihood of mother-to-child transmission is estimated to be approximately 3% if a mother with <16 copies/10^5^ cells in peripheral blood mononuclear cells breast-feeds^9, 10^. Thus, if the result of this confirmatory test is below detection limit (<4 copies/10^5^ cells), the percentage of mother-to-child transmission is considered to be <3% because the amount of virus is even lower. As a reference, the mother-to-child transmission rate of 3% has about the same risk as that of the mother-to-child transmission rate if mothers with confirmed HTLV-1 transmission opt for complete formula feeding^3, 8, 11^.

1. **Points to consider regarding insurance-covered care**

The HTLV-1 nucleic acid detection (PCR) method has been covered by insurance from April 1, 2018 for pregnant women with “indeterminate” results from LIA; however, the HTLV-1 nucleic acid detection (PCR) method is not yet covered in individuals other than pregnant women with “indeterminate” results from LIA.

**References**

1. Kamihira S, Yamano Y, Iwanaga M, et al. Intra- and inter-laboratory variability in human T-cell leukemia virus type-1 proviral load quantification using real-time polymerase chain reaction assays: a multi-center study. Cancer Sci. 2010;101(11):2361-7.
2. Health, Labour and Welfare Sciences Research Grant "Standardization of Diagnostic Methods for HTLV-1 Infections and Elucidation of Risks of Onset" Group (Representative: Isao Hamaguchi), 2011-2013 Comprehensive Research Report.
3. Manual for Prevention of HTLV-1 Mother-to-Child Transmission and Guidance on Health. Health, Labour and Welfare Sciences Special Research Project "Research on Standardization of Health Guidance for Prevention of HTLV-1 Mother-to-Child Transmission" Group (Representative: Hiroyuki Moriuchi), Fiscal Year 2010 Study Report.
4. Qiu X, Hodges S, Lukaszewska T, et al. Evaluation of a new, fully automated immunoassay for detection of HTLV-I and HTLV-II antibodies. J Med Virol. 2008;80(3):484-93.
5. Deguchi M, Kagita M, Yoshioka N, et al. Fundamental evaluation of six immunological assay reagents for HTLV antibody. Igaku to Yakugaku. 2011;66(6):1053-9.
6. Sonoyama S, Uno N, Okada Y, et al. Performance evaluation of the HTLV-1/2 antibody measurement reagent “Elecsys HTLV I/II.” Japanese Journal of Medical Technology. 2016;65(6):642-8.
7. Kuramitsu M, Sekizuka T, Yamochi T, et al. Proviral features of human T cell leukemia virus type 1 in carriers with indeterminate Western blot analysis results. J Clin Microbiol. 2017;55(9):2838-49.
8. Manual for Prevention of HTLV-1 Mother-to-Child Transmission. Health, Labour and Welfare Administrative Survey Project Grant and Research Project for the Next Generation of Fundamental Research, such as for overcoming growth diseases, etc. "Research on prevention of HTLV-1 mother-to-child transmission: Cohort study of infants born to pregnant women with positive HTLV-1 antibodies" Group (Representative: Kazuo Itabashi) Fiscal Year 2016 Study Report.
9. Li HC, Biggar RJ, Miley WJ, et al. Provirus load in breast milk and risk of mother-to-child transmission of human T lymphotropic virus type I. J Infect Dis. 2004;190(7):1275-8.
10. Biggar RJ, Ng J, Kim N, et al. Human leukocyte antigen concordance and the transmission risk via breast-feeding of human T cell lymphotropic virus type I. J Infect Dis. 2006;193(2):277-82.
11. Health, Labour and Science Grant-in-Aid for Scientific Research, Special Research Project "Research on prevention of HTLV-1 mother-to-child transmission" Group (Representative: Shigeru Saito) Fiscal Year 2009 Summary and Distributed Research Report (Guidance for Physicians).

Drafted on November 2019

**Appendix**

**Flowchart for the diagnosis of HTLV-1 infection**
